# Association between Exposure to Benzodiazepines and Related Drugs and Survivorship of Total Hip Replacement in Arthritis: A Population-Based Cohort Study of 246,940 Patients

**DOI:** 10.1371/journal.pone.0155783

**Published:** 2016-05-24

**Authors:** Dan Beziz, Sandrine Colas, Cédric Collin, Rosemary Dray-Spira, Mahmoud Zureik

**Affiliations:** Department of Epidemiology of Health Products, Division for Science and European Strategy, French National Agency for Medicines and Health Products Safety (ANSM), Saint-Denis, France; Queen's University, CANADA

## Abstract

**Background:**

Total hip replacement (THR) is successful in treating hip arthritis. Prosthetic survivorship may depend on the medications taken by the patient; particularly, the role of benzodiazepines and related drugs (Z-drugs) with THR revision has been poorly investigated. Our objective was to compare THR short-term survivorship according to level of exposure to benzodiazepine and Z-drugs.

**Design, Setting and Participants:**

All French patients aged 40 years or older, having undergone primary THR from January 1, 2009, through December 31, 2012, for arthritis according to French national health insurance databases were included in the cohort. Outcome of interest was THR revision, including any surgical procedure in which the implant or any component was changed or removed. Follow-up started the day the primary THR was performed. Observations were right-censored on December 31, 2014, if neither revision nor death had yet occurred. Exposure of interest was the cumulative defined daily doses per day (cDDD/day) of benzodiazepines and Z-drugs dispensed within 6 months before or after inclusion. We defined four exposure groups; cDDD/d = 0: unexposed; <0.08: low exposure;] 0.08–0.38]: medium exposure; >0.38: high exposure. THR survivorship was assessed according to level of exposure to benzodiazepines and Z-drugs in univariate and multivariate Cox models adjusted for patient, THR and implanting center characteristics.

**Results:**

The study cohort comprised 246,940 individuals: mean age at baseline, 69.9 years; women, 57.9%; unexposed: 51.7%; low exposure: 16.7%; medium exposure: 15.9%; and high exposure: 15.7%. During the median 45-month follow-up, 9043 individuals underwent prosthetic revision. Adjusted hazard ratios in low, medium and high exposed groups were 1.18 (95%CI, 1.12–1.26; P<0.001), 1.32 (95%CI, 1.24–1.40; P<0.001) and 1.37 (95%CI, 1.29–1.45; P<0.001), respectively, compared to unexposed.

**Conclusion and Relevance:**

Exposure to benzodiazepines and Z-drugs is associated with an increased risk of THR revision, with a dose-response relationship. Cautious prescribing might be needed as well as careful history examination and assessment of risk for patients with a hip prosthesis.

## Introduction

Osteoarthritis, a degenerative cartilage disease, is a leading cause of disability worldwide in the elderly [1–3]. Total hip replacement (THR) to replace a damaged coxo-femoral joint, is one of the most common orthopaedic procedures for treating all-aetiology hip osteoarthritis [4]. Primary or secondary degenerative osteoarthritis is the main indication [5], but THR is also used to treat rheumatoid arthritis or avascular necrosis or after trauma, common in elderly women especially. THR prevalence has substantially increased in industrialized countries in recent decades, largely due to population ageing. Despite positive results, revision is sometimes necessary (approximately 1% per year). Prosthetic revision is longer and more complex than primary arthroplasty and has a higher incidence of postsurgical complications. Hence, understanding factors associated with prosthetic survivorship constitutes a public health issue.

BZDs and related drugs (Z-drugs) are the most widely-used drug classes [6]. Their pharmacological properties multiply therapeutic applications in anxiety, insomnia, epilepsy and muscle spasms. Their use is a matter of concern among world public health regulators due to the risks from both short and long-term exposure, particularly in elderly patients [7] where BZDs are often prescribed inappropriately [8]: although the long-term effectiveness of benzodiazepines remains unproved for insomnia and questionable for anxiety [9,10], chronic use is predominant in older people. Several studies showed that BZDs consumption in France is among the highest in Europe [11]. Consumption is not devoid of adverse effects: memory problems, daytime sedation, ataxia, increased risk of motor vehicle accidents and falls [8].

Although the association between BZDs exposure and increased risk of hip fracture leading to THR is well-documented [12–23], few researches studied the association with THR revision.

The aim of this study was to examine the association between BZDs and Z-drugs exposure and THR short-term survivorship in a population-based cohort, using French health insurance system linked to hospital discharge databases [24–28].

## Material and Methods

### Data Sources

We used the French Health Insurance Information System (SNIIRAM), [24, 27–29], covering the entire French population, with different employment-based schemes. We only used the data for general scheme beneficiaries (approximately 77% of the population), for whom SNIIRAM records, with dates, outpatient drugs (Anatomical Therapeutic Classification codes), medical devices, services and procedures reimbursed. The database does not state medical indications per reimbursement but contains patients’ demographic, administrative, and medical details (chronic conditions such as diabetes mellitus, cancer or cardiovascular disease) and date of death. An anonymous, unique patient identifier links SNIIRAM information to national hospital discharge databases (Programme de Médicalisation des Systèmes d’Information, PMSI), providing reasons for admission (using International Statistical Classification of Diseases, 10^th^ Revision, ICD-10).

### Inclusion and Exclusion Criteria

**Fig 1:**
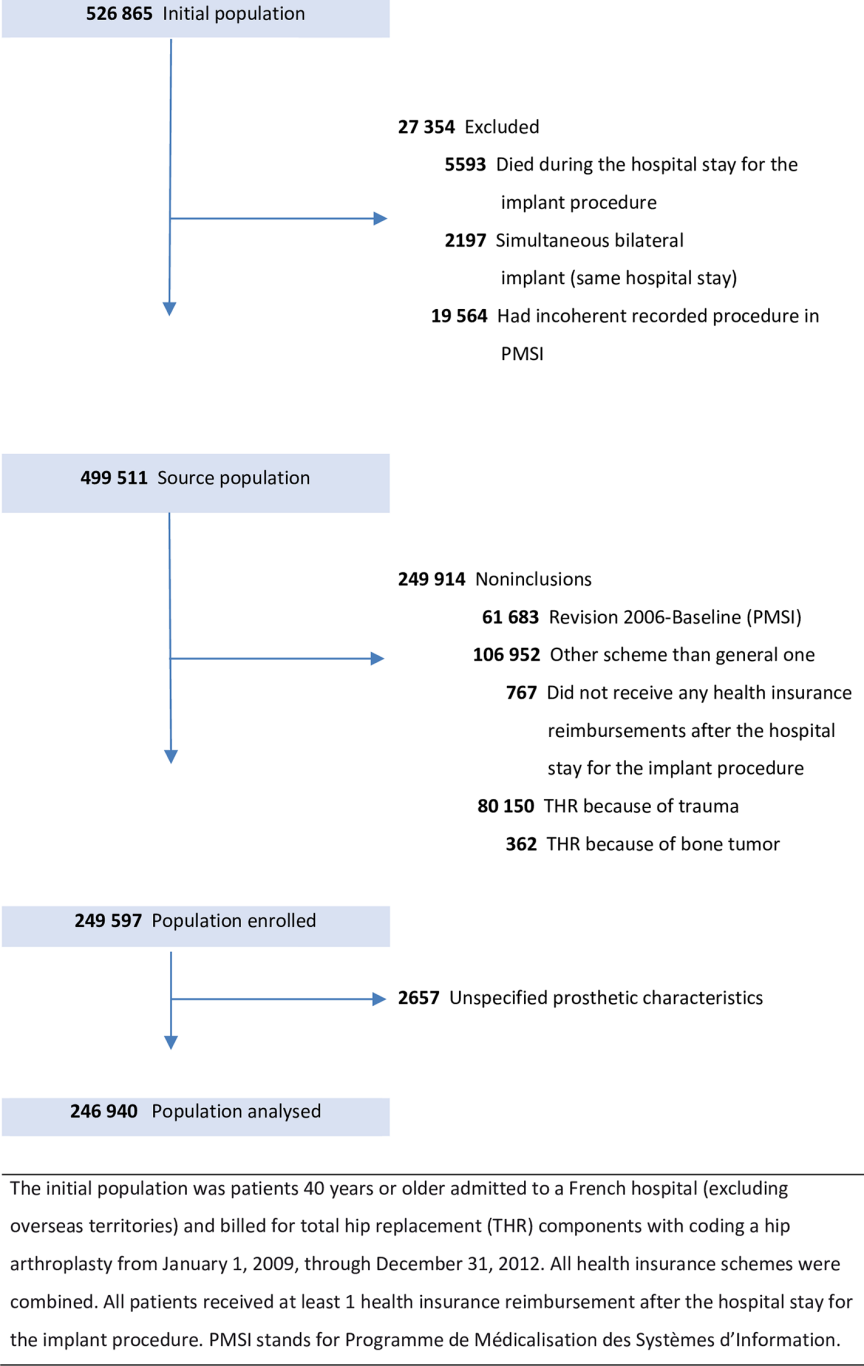
Study population flowchart.

The eligible population was patients aged 40 years or older, having undergone unilateral primary THR for osteoarthritis from January 1, 2009, through December 31, 2012 (48 months). Patients having primary THR for trauma or bone cancer, prosthetic revision before the inclusion period, simultaneous bilateral THR, not being reimbursed 6 months after THR or with non-matching procedure in PMSI were excluded. The entire study cohort population of 249,597 patients was described. THR characteristics were missing for 2,657, excluded from subsequent analyses, leaving 246,940 (**Fig 1**); 61,054 were enrolled in 2009, 60,808 in 2010, 62,230 in 2011 and 62,848 in 2012.

Approval was obtained from the French data protection agency (Commission Nationale de l’Informatique et des Libertés). Informed consent was not required as information was collected anonymously.

### Outcome and follow-up

Outcome was THR revision (including any surgical reintervention in which the implant or any components was changed or removed). Follow-up started at the date of primary THR. Observations were right-censored on December 31, 2014, if neither revision nor death had yet occurred. The minimum backward period was two years (maximum six years).

### Variable of Interest: Exposure to benzodiazepines and Z-drugs

All common BZDs and two Z-drugs (Zopiclone, Zolpidem) referred to as “BZDs”, were exposure of interest. We identified patients prescribed BZDs six months before or after inclusion, as baseline time-window of interest, to have a sufficient period to accurately measure exposure around implantation.

The defined daily dose (DDD), recommended by the World Health Organization, is the average dose per day for a drug used in its primary indication in adults and a unit used for measuring a prescribed drug amount [30]. We calculated the sum of the BZDs and Z-drugs DDDs dispensed (all drugs combined) during the time window of interest, divided by 365 (days), to get a cumulative DDD per day (cDDD/d), as an exposure indicator. We then definerd four exposure categories: three tertiles for exposed patients (Low = cDDD/d < 0.08; Medium = 0.08 ≤ cDDD/d ≤ 0.38; High = cDDD/d > 0.38) and the fourth category for unexposed.

### Covariates

We collected a series of patient, implantation centre and THR characteristics known to be or suspected of being associated with a risk of post-arthroplasty complications. Patients’ age, sex and date of death, came from the SNIIRAM database.

At baseline, treatments were identified by prescriptions (Anatomical Therapeutic Classification codes) reimbursed at least once within 180 days before or after inclusion, antidepressants, antihypertensives, oral corticosteroids, osteoporosis treatments, psychostimulants, antiepileptics, anxiolytic or hypnotic non-benzodiazepines (non-BZD), antipsychotics. Diabetes mellitus, obesity, Parkinson’s disease, immunodeficiency and chronic kidney disease were defined (ICD-10 categories) on the basis of hospital discharge reports or chronic condition recorded the year before inclusion with relevant prescriptions. The mean number of THR procedures per month (during the 4-years inclusion period) was calculated. Whether implantation centres were private or public and hospital stay (in days) were also collected.

Four types of THR fixation technique are available: uncemented, both-sides cemented, hybrid (femoral component cemented, acetabular component uncemented), and reverse hybrid (femoral component uncemented, acetabular component cemented). Four bearing couples were analyzed: ceramic-on-ceramic (CoC), ceramic-on-polyethylene (CoP), metal-on-metal (MoM) and metal-on-polyethylene (MoP) [31].

### Statistical Analysis

#### Main Analyses

Continuous variables were not normally distributed and were categorized. Patient, centre, hospital stay and THR (cement, bearing surface) characteristics were compared according to cDDD/d group using a Chi-square test. Kaplan-Meier survival analysis and log-rank test were used to assess differences in cumulative revision risk according to cDDD/d group. Hazard ratios (HRs) for revision according to cDDD/d groups were assessed using univariate and multivariate Cox proportional hazards regression models, adjusting for possible confounding factors: sex; age at implantation: young (40–59), middle-aged (60–74) and elderly (≥75 years); diabetes mellitus; obesity; Parkinson’s disease; immunodeficiency; medication (antidepressants, oral corticosteroids, antiosteoporotics, psychostimulants, non-BZD antiepileptics, anxiolytic/hypnotic non-BZD, antipsychotics); public or private; centre activity volume (tertiles); stay (3 groups: <6; 6–12; >12 days); cement type (4 groups); and bearing surface (4 groups).These characteristics were included simultaneously in the multivariate Cox proportional hazards regression model. Assumption of proportional hazards was graphically assessed for each variable. Interactions between exposure and age, as well as sex, in association with prosthetic survivorship were investigated.

#### Sensitivity Analyses

We performed several sensitivity analyses to challenge the robustness of our results.

We considered exposure to BZDs not in terms of cDDD/d, but as binary variable exposed/unexposed, fixed at baseline (same time-window of interest as the main analysis) in a multivariate Cox model adjusted for the same covariates as the main analysis, and studied the association between BZDs exposure and THR survivorship in each stratum of age (3 groups), sex (male/ female), exposure to antidepressants (Yes/No) and activity sector (public / private). As exposure to BZDs may vary over time, especially after THR, we considered exposure to BZDs as binary time-dependent variable. History of BZDs exposure was estimated by combining drug prescriptions and time to dispensing data: more than 40 days between two consecutives deliveries being an unexposed period. HRs for revision related to exposure to BZDs were then assessed using univariate and multivariate Cox proportional hazards regression models adjusting for the same factors as the main analysis, except exposure to antidepressants, which here was also treated as time-dependant binary variable. To complete these analyses with time-dependent exposure to BZDs, we estimated and described mean cDDD/d over time during follow-up in exposed patients.

We assessed anxiolytic-BZDs and hypnotic-BZDs exposure separately, within 6 months before or after inclusion. HRs for revision following anxiolytic-BZDs alone, hypnotic-BZDs alone and both anxiolytic-and hypnotic-BZDs were assessed using univariate and multivariate Cox proportional hazards regression models. Diazepam, oxazepam, clorazepate, chlordiazepoxide, clonazepam, bromazepam, clobazam, lorazepam, alprazolam, loflazepate and clotiazepam as anxiolytic-BZDs and nitrazepam, flunitrazepam, estazolam, triazolam, lormetazepam, temazepam, midazolam, loprazolam, Zopiclone and Zolpidem as hypnotic-BZDs.

Statistical tests were 2-tailed with 5% alpha-risk and analyses were performed using SAS Base 9.4 and SAS Enterprise Guide 4.3 software (SAS Institute Inc).

## Results

### Baseline

Mean age was 69.9 years (Standard Deviation, SD: 10.8). Women (57.9% of enrolled individuals) were significantly older than men (71.6 vs. 67.5, P < .0001). Implantation was performed in a private hospital in 65.7% of cases and nearly half of patients were implanted in centres performing 14 to 38 monthly procedures (**Table 1**). Median hospital stay was 8 days (interquartile range, IQR: 7–10). Fixation was uncemented: 73.0%, cemented: 6.1%, hybrid: 19.2% and reverse hybrid: 1.6%. Bearing surfaces were CoC: 40.7%, MoP: 34.0%, CoP: 21.0% and MoM: 3.3%. Regarding comorbidities, 9.4% were obese, 5% had dementia and 2.5% Parkinson’s disease. Regarding treatments, 18.6% were exposed to antidepressants (significant difference between men and women, 11.7% vs. 23.6%, P < .0001), 27.9% to oral corticosteroids, 8.1% to antiepileptic drugs (non-BZD) and 12.9% to anxiolytic or hypnotic drugs, non-BZD.

**Table 1:** Baseline Characteristics of enrolled patients, Hospital Stays and THR according to sex.

		Total	Male	Female
		(n = 249 597)	(n = 105 150)	(n = 144 447)
		%	%	%
**Patient Characteristics**				
Sex	Male	42.1	100.0	
	Female	57.9		100.0
Age category, y	40–59	17.7	23.7	13.3
	60–74	44.4	46.8	42.7
	≥ 75	37.9	29.5	44.0
**Comorbidities**	** **	** **	** **	** **
Diabetes mellitus		11.9	14.5	10.0
Obesity		9.4	9.9	9.0
Parkinson's disease		2.5	2.2	2.8
Immunodeficiency		1.4	1.2	1.6
Cancer		2.3	2.5	2.1
Asthma or COPD		15.2	16.3	14.4
Dementia		5.0	2.8	6.6
Chronic kidney disease		1.2	1.3	1.2
**Treatments**				
Antidepressant		18.6	11.7	23.6
Antihypertensive		60.5	59.6	61.3
Oral corticosteroid		27.9	25.2	29.8
Antiosteoporotics		10.8	2.0	17.2
Psychostimulant		0.8	0.6	1.0
Antiepileptic (non-BZD)		8.1	7.3	8.8
Anxiolytic/Hypnotic (non-BZD)		12.9	10.9	14.4
Antipsychotic		4.0	3.8	4.1
**Center and Hospital stay Characteristics**				
Activity sector	Public	34.3	34.3	34.4
** **	Private	65.7	65.7	65.6
No. of procedures per month	< 14	25.1	25.2	25.1
	14–38	49.9	50.3	49.6
	> 38	25.0	24.6	25.4
Hospital stay Duration (days)	<6	5.7	6.9	4.9
	6–12	84.2	84.5	84.0
	>12	10.1	8.6	11.1
**THR Characteristics**				
THR Cement Type	Uncemented	73.0	78.6	69.0
	Cemented	6.1	4.4	7.4
	Hybrid	19.2	15.9	21.6
	Reverse Hybrid	1.6	1.1	2.0
THR bearing surface	CoC	40.7	46.8	36.3
	CoP	21.0	20.0	21.7
	MoM	3.3	4.4	2.5
	MoP	34.0	28.0	38.4
	Unspecified	1.1	0.9	1.2

Abbreviations: CoC, ceramic-on-ceramic; CoP, ceramic-on-polyethylene; MoM, metal-on-metal; MoP,metal-on-polyethylene; THR, total hip replacement.

In our population, 48.3% of subjects were exposed to BZDs (15.7% highly exposed): 17.5% were exposed to anxiolytic-BZDs, 14.8% to hypnotic-BZDs, 9.8% to both anxiolytic and hypnotic-BZDs and 6.2% to other BZDs, such as an antiepileptics or muscle relaxants. Exposed patients received a mean cDDD/d of 0.40; they received an average 5.4 BZDs deliveries within the year around inclusion.

### Baseline Characteristics according to BZDs exposure level

Table 2 shows baseline patient, hospital stay and THR characteristics according to cDDD/d group. Women and elderly patients were more exposed to BZDs than men and younger patients, respectively. Among exposed patients, median age was 72 years (IQR: 63–79) and 64.8% were women, and it was 70 years (IQR: 62–78) and 51.3% respectively among unexposed subjects. Patients with Parkinson’s disease were more often exposed to BZDs (27.3% high cDDD/d) than patients without this condition. We did not find major differences in BZDs exposure between diabetics / non-diabetics and obese / non-obese subjects. Patients taking antidepressants, antipsychotics, antiepileptics or anxiolytic/hypnotic non-BZD were more often exposed to BZDs: 77.8% of patients taking antidepressants were exposed to BZDs (39.2% highly exposed) versus 41.5% of patients not taking antidepressants (10.3% highly exposed). Patients staying longer in hospital after THR had higher BZDs exposure.

**Table 2:** Characteristics of the patients, centers, hospitals Stays and THR, according to exposure to BZDs.

		Unexposed, %	Exposed, %		
			Low	Medium	High
		(n = 127 797)	(n = 41 196)	(n = 39 181)	(n = 38 766)
**Patient Characteristics**					
Sex	Male	59.7	16.5	12.8	11.0
	Female	45.9	16.8	18.1	19.1
Age category, y	40–59	53.2	19.2	14.3	13.4
	60–74	53.5	17.1	14.8	14.6
	≥ 75	49.0	15.0	17.9	18.1
**Comorbidities**					
Diabetes mellitus	No	51.9	16.9	16.0	15.3
	Yes	50.8	15.4	15.1	18.7
Obesity	No	51.8	16.8	16.0	15.5
	Yes	51.7	16.0	14.7	17.6
Parkinson's disease	No	52.2	16.7	15.8	15.4
	Yes	35.7	16.5	20.3	27.6
Immunodeficiency	No	51.8	16.7	15.9	15.7
	Yes	49.0	17.0	17.2	16.9
Chronic kidney disease	No	51.8	16.7	15.8	15.6
	Yes	47.4	14.8	17.7	20.1
**Treatments**					
Antidepressant	No	58.5	17.2	13.9	10.3
	Yes	22.2	14.3	24.3	39.2
Antihypertensive	No	56.1	18.1	14.2	11.6
	Yes	48.9	15.8	17.0	18.4
Oral corticosteroid	No	55.6	15.7	14.6	14.2
	Yes	41.9	19.3	19.2	19.7
Antiosteoporotics	No	53.0	16.6	15.4	15.0
	Yes	41.6	17.2	20.0	21.2
Psychostimulant	No	51.9	16.7	15.8	15.6
	Yes	38.4	16.7	19.2	25.7
Antiepileptic (non-BZD)	No	53.3	16.5	15.4	14.5
	Yes	31.6	18.5	20.8	29.1
Anxiolytic/Hypnotic (non-BZD)	No	54.6	16.4	15.1	13.9
	Yes	32.5	18.4	20.9	28.2
Antipsychotic	No	52.9	16.9	15.7	14.5
	Yes	25.3	11.8	19.1	43.8
**Center and hospital stay characteristics**					
Activity sector	Public	50.8	16.4	16.1	16.6
	Private	52.2	16.8	15.7	15.2
No. of procedures per month	< 14	50.5	16.5	16.5	16.6
	14–38	51.9	16.6	15.7	15.8
	> 38	52.8	17.1	15.5	14.6
Duration in days	< 6	59.9	16.0	12.9	11.3
	6–12	52.1	16.8	15.7	15.4
	> 12	44.2	15.7	19.2	20.9
**THR Characteristics**					
THR Cement Type	Uncemented	52.7	16.8	15.4	15.1
	Cemented	48.1	16.2	17.9	17.8
	Hybrid	49.6	16.5	16.7	17.3
	Reverse Hybrid	46.3	15.5	19.1	19.1
THR bearing surface	CoC	54.3	17.6	14.7	13.4
	CoP	51.2	16.3	16.1	16.4
	MoM	52.4	19.0	15.0	13.6
	MoP	49.0	15.6	17.2	18.1

Exposure groups: Low:] 0–0.08]; Medium:] 0.08–0.38]; High: > 0.38 cDDD/d of BZDs within 6 months before or after inclusion

### Revision and BZDs exposure relationship

**Fig 2:**
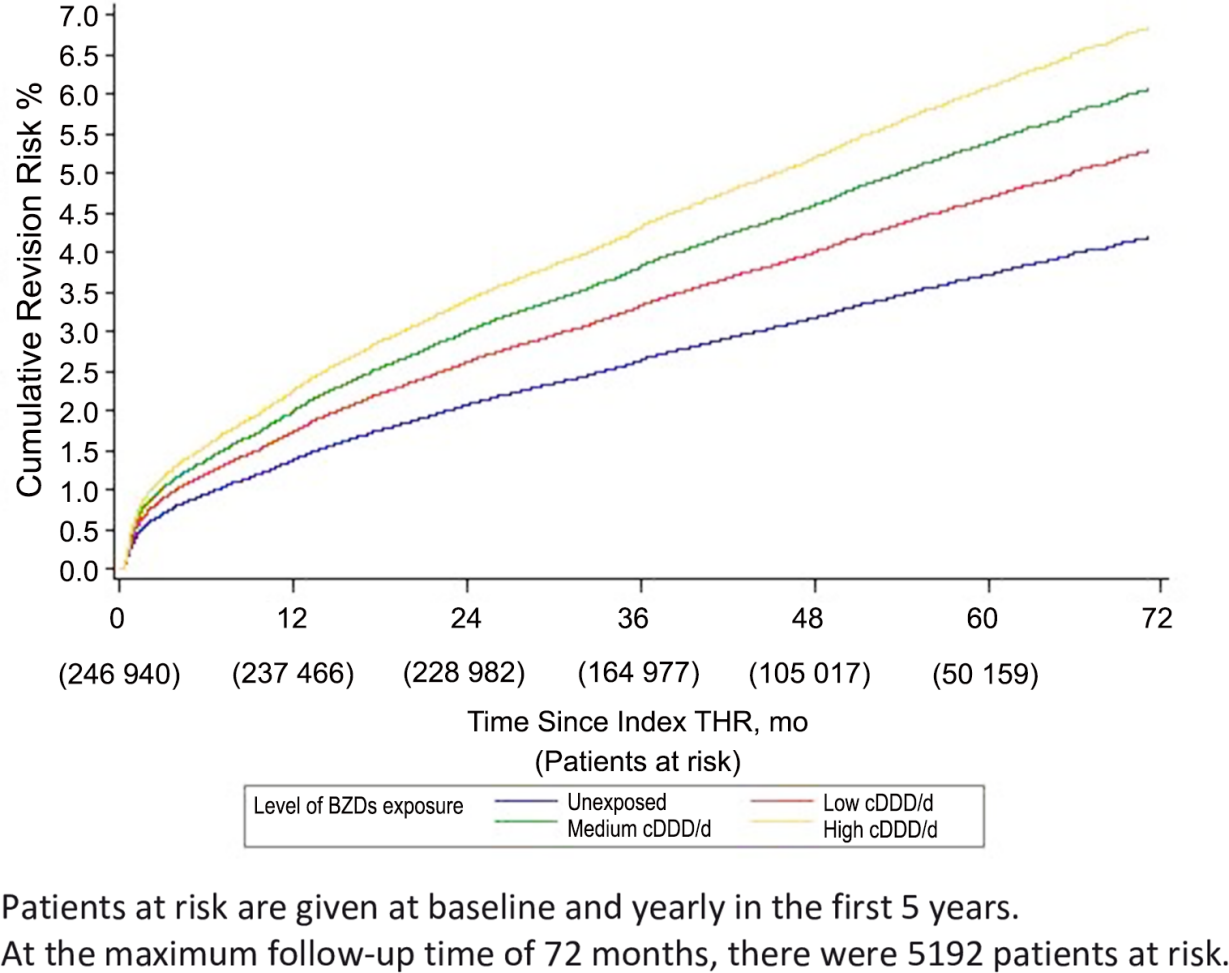
Kaplan Meier Cumulative Revision Risk According to Level of Exposure to BZDs.

During 45 months follow-up (IQR: 33–58; range: 0.03–72), 9,043 prosthetic revisions (3.7%) were recorded. Univariate survival analysis showed exposure to BZDs to be associated with a significant increase in revision risk compared with unexposed patients, with a dose-response relationship (cumulative revision rates: 3.0% in unexposed, 3.9% in low doses, 4.4% in medium doses and 4.8% in high doses of BZDs) (**Fig 2**). Univariate and Multivariate analyses gave similar results: adjusted HR, aHR = 1.18 (95%CI: 1.12–1.26) for low cDDD/d, aHR = 1.32 (95%CI: 1.24–1.40) for medium cDDD/d and aHR = 1.37 (95%CI: 1.29–1.45) for high cDDD/d, compared to unexposed patients. (**Table 3**).

**Table 3:** Associations between exposure level to BZDs and prosthetic revision.

		Revisions	Univariate Cox Model		Multivariate Cox Model	
		(n = 9043)	(N = 246 940)		(N = 246 940)	
	N	%	Crude HR (IC 95%)	p-value	Adjusted HR^§^ (IC 95%)	p-value
Unexposed	127797	3.0	ref		ref	
Low	41196	3.9	1.26 (1.19–1.34)	<0.0001	1.18 (1.12–1.26)	<0.0001
Medium	39181	4.4	1.45 (1.37–1.53)	<0.0001	1.32 (1.24–1.40)	<0.0001
High	38766	4.8	1.63 (1.55–1.73)	<0.0001	1.37 (1.29–1.45)	<0.0001

§ Hazard Ratios were adjusted for: sex, age at implantation, diabetes mellitus, obesity, Parkinson's disease, immunodeficiency, exposure to antidepressant, oral corticosteroid, antiosteoporotics, psychostimulant, antipsychotic, antiepileptic (non-BZD), anxiolytic or hypnotic (non-BZD), public or private sector, center volume of activity, duration of stay, cement type and bearing surface.

In univariate and multivariate Cox proportional hazards regression analyses, prosthetic survivorship was also significantly associated with sex, age, obesity: female and older patients having a better prosthetic prognosis than men and younger patients, respectively. Parkinson’s disease and exposure to antidepressant, oral corticosteroid, antiepileptic and anxiolytic or hypnotic non-BZD are risk factors for THR revision, as are uncemented prosthesis, MoM THR and long stay. Complete results are provided in S1A Table.

No significant cDDD/d and sex or age interactions were detected in the relationship with prosthetic survivorship.

### Sensitivity Analyses

The first sensitivity analysis showed aHR for BZDs-exposed patients at baseline, compared to unexposed to be consistent with the main analysis: 1.25 (95%CI: 1.19–1.31), as were results for stratified analyses on sex, age, exposure to antidepressants and activity: aHRs range 1.22 to 1.27. Sensitivity analysis results with BZDs exposure and exposure to antidepressant drugs as time-dependant variables were also consistent with the main analysis results: aHR 1.29 (95%IC: 1.23–1.36) for BZDs-exposed patients, versus unexposed (**Table 4)**. During follow-up, cDDD/d was 0.37 for over-time exposed patients; they received 4.2 BZDs average yearly deliveries.

**Table 4:** Sensitivity analyses–Stratified analyses (with exposure to BZDs studied as binary factor).

			Adjusted HR^§^ (95% CI)		P Value
		No. of Patients	Unexposed	Exposed to BZDs	
**Overall**					
**Baseline (fixed) exposure to BZDs**		246940	1 (ref)	1.25 (1.19–1.31)	<0.0001
**Time-dependent exposure to BZDs**		246940	1 (ref)	1.29 (1.23–1.36)	<0.0001
**Analyses stratified on**					
**Sex**	Male	104217	1 (ref)	1.24 (1.16–1.33)	<0.0001
	Female	142723	1 (ref)	1.25 (1.18–1.33)	<0.0001
**Age group**	40–59	43748	1 (ref)	1.27 (1.16–1.40)	<0.0001
	60–74	109991	1 (ref)	1.25 (1.17–1.34)	<0.0001
	≥ 75	93201	1 (ref)	1.22 (1.13–1.31)	<0.0001
**Exposure to antidepressant**	No	201142	1 (ref)	1.24 (1.18–1.31)	<0.0001
	Yes	45798	1 (ref)	1.25 (1.12–1.39)	<0.0001
**Hospital sector**	Public	83925	1 (ref)	1.26 (1.16–1.35)	<0.0001
	Private	163015	1 (ref)	1.24 (1.18–1.31)	<0.0001
**Analyses according to**					
**class of BZDs exposure at baseline**					
**Exposure to Anxiolytic BZDs only**		43128	1 (ref)	1.23 (1.16–1.30)	<0.0001
**Exposure to Hypnotic BZDs only**		36402	1 (ref)	1.28 (1.21–1.36)	<0.0001
**Exposure to both BZDs**		24163	1 (ref)	1.37 (1.28–1.47)	<0.0001
**Exposure to other class of BZDs**		16519	1 (ref)	1.28 (1.18–1.39)	<0.0001

§ Hazard Ratios were adjusted for: sex, age at implantation, diabetes mellitus, obesity, Parkinson's disease, immunodeficiency, exposure to antidepressant, oral corticosteroid, antiosteoporotics, psychostimulant, antipsychotic, antiepileptic (non-BZD), anxiolytic or hypnotic (non-BZD), public or private sector, center activity, duration of stay, cement type and bearing surface, in multivariate Cox models.

When assessing separately baseline exposure to anxiolytic-BZDs alone, exposure to hypnotic-BZDs alone and exposure to both BZDs, aHR was 1.23 (95%IC: 1.16–1.30) for anxiolytic-BZDs, 1.28 (95%IC: 1.21–1.36) for hypnotic-BZDs and 1.37 (1.28–1.47) for both, (p<0.0001).

## Discussion

In this population-based study including all THRs performed in France because of arthritis, patients exposed to BZDs had a significantly poorer THR survivorship than unexposed patients, with dose-effect relationship, independently of other prosthetic revision risk factors. Similar findings were found when stratifying analyses on sex, on age group, on hospital sector or on exposure to antidepressant drugs, ensuring the robustness of our results. This association was seen regardless of BZDs category, with increased risk from concomitant anxiolytic-BZD and hypnotic-BZD use. To our knowledge, this is the first study which examined the association between THR revision risk and level of exposure to BZDs in a population-based cohort. Our conclusions may not apply to patients having THR for trauma or bone cancer, these being excluded from our study.

The event analyzed was short-term prosthetic revision, main indications for which are osteoarticular infection, dislocation and periprosthetic fracture [32, 33]. BZDs are not known to affect bone quality or growth, or to increase infection rate. A major known adverse effect of BZDs is oversedation, which includes symptoms such as drowsiness, poor concentration and vigilance, ataxia, dysarthria, diplopia, myasthenia, vertigo and mental confusion. In the elderly, oversedation is more marked and persistent: even when taken at night, even low-dose hypnotic sedatives may cause acute confusional states the next day (partly due to decreased metabolism); some authors have shown increased risk of falling in elderly [34, 35]. Previous works studying the association between BZDs and risk of hip fracture found BZDs exposure to be a risk factor: aHRs 1.2 to 1.4 [12, 36, 37]. Consequently, it seems reasonable to assume that in exposed patients, BZDs may increase the risk of falls, possibly leading to THR fracture and revision; it is our hypothesis. Nonetheless, we were unable to identify direct causes for revision; studies covering both BZDs exposure and indications for revision would be necessary to investigate a possible underlying mechanism.

In our study, we measured baseline BZDs exposure in terms of cDDD/d. The DDD was first constructed as a technical measure to estimate specific drug consumption per capita, based on aggregate sales data records. It is nonetheless an internationally recognized measure which enables dosage comparisons between drug categories, making it the first choice for comparing prevalence of use and doses, over time and between areas [15,18,38]. Our assessment of baseline BZDs exposure as fixed factor in the main analysis could also be discussed, as exposure may vary overtime, especially after THR. However, estimated mean cDDD/d for patients exposed at baseline was very close to that of patients exposed during follow-up: cDDD/d were 0.40 and 0.37 respectively. We also compared the average number of times BZDs were dispensed within a year both for patients exposed at baseline and during follow-up: they respectively received BZDs 5 and 4 times yearly on average. This suggests a similar pattern in baseline exposure to BZDs and exposure to BZDs during follow-up. We also conducted sensitivity analyses where BZDs exposure was a fixed binary variable and then binary time-dependent variable; the results were both similar to those of the main analysis.

Regarding the proportion of BZDs-exposed patients, our results are consistent with data available for the elderly French population [11, 39–41], with almost every other patient over 70 being exposed. Similarly, we found that 64.8% of patients exposed were women compared to 64.2% in the last report by the French National Agency for Medicines and Health Products Safety [41]. Average cDDD/d in exposed patients from our cohort was higher than that in the general French population. This is consistent with the fact that patients from our cohort had arthritis, which causes pain, were older and mainly women; age and sex are known to be associated with exposure [11]. The 3.7% prosthetic revision rate at 45 months follow-up is consistent with data from international registries [32, 42–47]. Our results for the covariates studied also concord with previous findings which identify younger age [48–51], male sex [47,48], obesity [52], uncemented prostheses [31,49], MoM THRs [31] and long hospital stay as primary THR revision risk factors. Low surgeon activity volume was also cited as a risk factor for early revision [53,54]; we found the same with hospital volume activity as proxy of that of surgeons. Regarding oral corticosteroid exposure, our results are also consistent with literature: some studies found patients taking corticosteroids chronically at increased risk of readmission, implant failure and post-THR infection [55–60].

The large number of patients, the possibility of comparing prosthetic survivorship according to BZDs exposure in cDDD/d and of adjusting survival analysis for many known and suspected prosthetic revision risk factors in multivariate Cox models are strengths of the study. Like all studies using healthcare databases, exposure to treatment is derived from drug dispensing data instead of effective drug intake. However, the possible classification bias is non-differential and given that BZDs dispensations are regularly renewed overtime, it is reasonable to assume that the drug is ingested.

Due to missing THR characteristics, we excluded 2,657 individuals (1.1%) from the analyses. In this group, revision rate was 3.8% and BZDs exposure 51.6% which is comparable to those in the analyzed individuals. We do not think excluding these patients substantially affected the observed associations.

Implant survivorship may not be the only relevant outcome after THR. Although prosthetic revision is an unambiguous outcome, it is not the only sign of implant failure; considering revision as the only outcome in implant survivorship has been debated in orthopaedic literature. Other markers, including quality of life and/or functional improvement, could be used as secondary outcome to identify non-revised implant failure, but the SNIIRAM database does not provide such information. Although direct costs related to BZDs are not an economic priority [39], their misuse and/or overuse result in significant indirect costs which is not our topic, but could be assessed by dedicated medico economic studies. Lastly, despite the large number of covariates taken for adjustment in the multivariate Cox model, residual confounding due to factors not collected in our study (for being absent from the data base, such as smoking history, alcohol ingestion, socioeconomic factors or premorbid nutrition …) cannot be ruled out.

## Conclusion

People who are prescribed BZDs have a higher THR revision rate. Cautious prescribing might be needed as well as careful history examination and assessment of risk for patients with a hip prosthesis.

## Supporting Information

S1 Table Associations between characteristics of the patients, hospital stays and THR with Prosthetic Revision. (DOCX)
