# Supplementary material for: Association between Exposure to Benzodiazepines and Related Drugs and Survivorship of Total Hip Replacement in Arthritis: A Population-Based Cohort Study of 246,940 Patients
Source: PLoS One. 2016 May 24;11(5):e0155783. doi: 10.1371/journal.pone.0155783 (PMC4878771; doi:10.1371/journal.pone.0155783)
Supplement: S1 Table — (DOCX) [file pone.0155783.s001.docx]

**S1 Table A. Associations between characteristics of the patients, hospital stays and THR with Prosthetic Revision.**

|  |  |  | **Revision** | **Univariate Cox model** | | **Multivariate Cox model** | |
| --- | --- | --- | --- | --- | --- | --- | --- |
|  |  |  | **(n=9043)** | **(N=246 940)** |  | **(N=246 940)** |  |
|  |  | **N** | **%** | **HRb (IC 95%)** | **p-value** | **HRa**^§^ (**IC 95%)** | **p-value** |
| **Patient Characteristics** |  |  |  |  |  |  |  |
| Sex | Male | 104217 | 3.7 | ref |  | ref |  |
|  | Female | 142723 | 3.6 | 0.96 (0.92-1.00) | 0.0540 | 0.93 (0.89-0.97) | 0.0008 |
| Age category, y | 40-59 | 43748 | 4.6 | ref |  | ref |  |
|  | 60-74 | 109991 | 3.6 | 0.80 (0.76-0.84) | <0.001 | 0.84 (0.79-0.88) | <0.001 |
|  | ≥ 75 | 93201 | 3.3 | 0.74 (0.70-0.78) | <0.001 | 0.79 (0.74-0.84) | <0.001 |
| **Comorbidities** |  |  |  |  |  |  |  |
| Diabetes mellitus | No | 217482 | 3.7 | ref |  | ref |  |
|  | Yes | 29458 | 3.8 | 1.06 (0.99-1.13) | 0.0800 | 1.02 (0.96-1.09) | 0.5721 |
| Obesity | No | 223721 | 3.6 | ref |  | ref |  |
|  | Yes | 23219 | 4.2 | 1.19 (1.11-1.27) | <0.001 | 1.13 (1.06-1.21) | 0.0002 |
| Parkinson's disease | No | 240791 | 3.6 | ref |  | ref |  |
|  | Yes | 6149 | 5.1 | 1.45 (1.30-1.62) | <0.001 | 1.27 (1.13-1.42) | <0.001 |
| Immunodeficiency | No | 243395 | 3.7 | ref |  | ref |  |
|  | Yes | 3545 | 4.6 | 1.28 (1.10-1.49) | 0.0018 | 1.12 (0.95-1.32 | 0.1653 |
| **Treatments** |  |  |  |  |  |  |  |
| Antidepressant | No | 201142 | 3.3 | ref |  | ref |  |
|  | Yes | 45798 | 5.2 | 1.59 (1.52-1.66) | <0.001 | 1.32 (1.25-1.39) | <0.001 |
| Oral corticosteroid | No | 178180 | 3.5 | ref |  | ref |  |
|  | Yes | 68760 | 4.2 | 1.24 (1.19-1.29) | <0.001 | 1.10 (1.05-1.16) | <0.001 |
| Antiosteoporotics | No | 220364 | 3.6 | Ref |  | ref |  |
|  | Yes | 26576 | 3.9 | 1.06 (0.99-1.13) | 0.1043 | 1.05 (0.98-1.13) | 0.1440 |
| Psychostimulant | No | 244942 | 3.7 | ref |  | ref |  |
|  | Yes | 1998 | 4.6 | 1.20 (0.98-1.47) | 0.0855 | 1.10 (0.90-1.36) | 0.3509 |
| Antiepileptic (non-BZD) | No | 226876 | 3.5 | ref |  | ref |  |
|  | Yes | 20064 | 5.5 | 1.63 (1.53-1.74) | <0.001 | 1.22 (1.13-1.32) | <0.001 |
| Anxiolytic/Hypnotic (non-BZD) | No | 215149 | 3.5 | ref |  | ref |  |
|  | Yes | 31791 | 5.1 | 1.51 (1.43-1.60) | <0.001 | 1.18 (1.11-1.26) | <0.001 |
| Antipsychotic | No | 237117 | 3.6 | ref |  | ref |  |
|  | Yes | 9823 | 5.3 | 1.54 (1.41-1.68) | <0.001 | 1.07 (0.98-1.18) | 0.1489 |
| **Center and hospital stay characteristics** |  |  |  |  |  |  |  |
| Activity sector | Public | 83925 | 3.8 | ref |  |  |  |
|  | Private | 163015 | 3.6 | 0.92 (0.88-0.96) | 0.0002 | 0.92 (0.88-0.96) | 0.0001 |
| No. of procedures per m | 14-38 | 123080 | 3.6 | ref |  | ref |  |
|  | < 14 | 61910 | 4.1 | 1.14 (1.09-1.20) | <0.001 | 1.11 (1.06-1.16) | <0.001 |
|  | > 38 | 61950 | 3.3 | 0.93 (0.88-0.98) | 0.0037 | 0.93 (0.88-0.98) | 0.0053 |
| Duration, d | 6-12 | 208136 | 3.6 | ref |  | ref |  |
|  | <6 | 14154 | 3.5 | 1.03 (0.94-1.13) | 0.5674 | 1.05 (0.96-1.15) | 0.3250 |
|  | >12 | 24650 | 4.2 | 1.20 (1.13-1.28) | <.001 | 1.14 (1.06-1.21) | 0.0002 |
| **THR characteristics** |  |  |  |  |  |  |  |
| THR Cement Type | Uncemented | 181174 | 3.9 | ref |  | ref |  |
|  | Cemented | 14307 | 3.0 | 0.75 (0.68-0.83) | <0.001 | 0.75 (0.68-0.82) | <0.001 |
|  | Reverse hybrid | 3761 | 4.7 | 1.26 (1.08-1.46) | 0.0026 | 1.18 (1.02-1.37) | 0.0311 |
|  | Hybrid | 47698 | 2.8 | 0.72 (0.68-0.76) | <0.001 | 0.74 (0.70-0.79) | <0.001 |
| THR bearing surface | CoC | 101537 | 3.9 | ref |  | ref |  |
|  | CoP | 52328 | 3.5 | 0.91 (0.86-0.96) | 0.0006 | 0.96 (0.91-1.02) | 0.1935 |
|  | MoM | 8156 | 4.9 | 1.16 (1.04-1.28) | 0.0058 | 1.17 (1.06-1.30) | 0.0026 |
|  | MoP | 84919 | 3.4 | 0.89 (0.85-0.93) | <0.001 | 0.95 (0.90-1.01) | 0.0745 |

§ Hazard Ratios were also adjusted for the cumulative Defined Daily Doses per day (cDDD/d) of BZDs in the 6 months before or after the index THR.

Abbreviations: CoC, ceramic-on-ceramic; CoP, ceramic-on-polyethylene; MoM, metal-on-metal; MoP, metal-on-polyethylene; THR, total hip replacement.
